# Longitudinal adherence to antiretroviral drugs for preventing mother-to-child transmission of HIV in Zambia

**DOI:** 10.1186/s12884-015-0697-7

**Published:** 2015-10-12

**Authors:** Sumiyo Okawa, Mable Chirwa, Naoko Ishikawa, Henry Kapyata, Charles Yekha Msiska, Gardner Syakantu, Shinsuke Miyano, Kenichi Komada, Masamine Jimba, Junko Yasuoka

**Affiliations:** Department of Community and Global Health, Graduate School of Medicine, The University of Tokyo, Tokyo, Japan; Chongwe District Community Health Office, Chongwe, Zambia; Ministry of Health Zambia-Japan International Cooperation Agency SHIMA project, Lusaka, Zambia; National Center for Global Health and Medicine, 1-21-1 Toyama, Shinjuku-ku, Tokyo 162-8655 Japan; Ministry of Health, Lusaka, Zambia

**Keywords:** Prevention of mother-to-child transmission of HIV (PMTCT), Antiretroviral (ARV), Adherence, Pregnancy, Zambia

## Abstract

**Background:**

Adherence to antiretroviral (ARV) drugs is essential for eliminating new pediatric infections of human immunodeficiency virus (HIV). Since the Zambian government revised the national guidelines based on option A (i.e., maternal zidovudine and infant ARV prophylaxis) of the World Health Organization’s 2010 guidelines, no studies have assessed adherence to ARVs during pregnancy up to the postpartum period. This study aimed to examine adherence to ARVs and identify the associated risk factors.

**Methods:**

A prospective cohort study was conducted in the Chongwe district from June 2011 to January 2014. Self-reported adherence to ARVs was examined during pregnancy and at one week, six weeks, and 24 weeks postpartum among 321 HIV-positive women. The probability of remaining adherent to ARVs was estimated using the Kaplan-Meier method, and the risk factors for non-adherence were identified using the Cox proportional hazard regressions—treating loss to follow-up as non-adherence. The statuses of HIV in HIV-exposed infants were assessed in January 2014.

**Results:**

During the study period, 326 infants were born to HIV-positive women, 262 (80.4 %) underwent HIV testing, and 11 (3.4 %) had their HIV infection detected at the time that they had the latest HIV testing as of January 2014. The ARV adherence rate was 82.5 % during pregnancy, 84.2 % at one week postpartum, 81.5 % at six weeks postpartum, and 70.5 % at 24 weeks postpartum. The probability of remaining adherent to ARVs was 0.61 at day 50, 0.35 at day 100, 0.18 at day 200, and 0.06 at day 300. Attending a referral health center (HC) was a risk factor for non-adherence compared with attending rural HCs that provided HIV care/treatment (adjusted hazard ratio [aHR] 0.71, 95 % confidence interval [CI] 0.57–0.88) and those that did not provide HIV care/treatment (aHR 0.58, 95 % CI 0.46–0.74). A new diagnosis of HIV infection compared to a known HIV-positive status before pregnancy was another risk factor for non-adherence (aHR 1.24, 95 % CI 1.03–1.50).

**Conclusions:**

Maintaining adherence to ARVs through pregnancy to the postpartum period remains a crucial challenge in Zambia. To maximize the treatment benefits, adherence to ARVs and retention in care should be improved at all health facilities.

## Background

The global epidemic of human immunodeficiency virus (HIV) has affected the lives of millions of children. In 2013, about 240,000 children were newly infected with HIV, with the majority of pediatric HIV infections being attributed to mother-to-child transmission [1]. In an attempt to eliminate new pediatric HIV infections, the World Health Organization (WHO) revised the guidelines for preventing mother-to-child transmission of HIV (PMTCT) in 2010 [2].

The guidelines recommended two options for antiretroviral (ARV) prophylaxis. In option A, pregnant women begin zidovudine (AZT) at 14 gestational weeks and continue it during pregnancy, while their infants receive nevirapine (NVP) throughout the breastfeeding period. In option B, pregnant women receive triple ARV prophylaxis from 14 weeks of pregnancy to the end of the breastfeeding period, while their infants receive NVP for the first four to six weeks. In both options, lifelong antiretroviral therapy (ART) is recommended for all women with a CD4 cell count ≤350 cells/mm^3^ or those in the WHO clinical stage 3 or 4. It is expected that ARV prophylaxis can reduce the risk of HIV transmission from 35 to 5 % among breastfeeding infants [2]. In 2013, WHO released new consolidated guidelines on the use of ARVs, in which two options for PMTCT, option B and B+ were recommended [3]. In option B+, all women initiate lifelong triple ARVs regardless of their WHO clinical stage or CD4 cell count [3].

Optimal adherence to ARVs is essential to protect children from acquiring HIV, maintain maternal health, and minimize the risk of drug resistance [4]. According to a meta-analysis on ARV adherence in the PMTCT program, 74 % of pregnant women in low-, middle-, and high-income countries achieved adequate adherence, and the ARV adherence rate was higher during pregnancy than the postpartum period [5].

In sub-Saharan Africa, only a few studies have assessed ARV adherence during pregnancy and the postpartum period [6–13]. In these studies, disclosure of the HIV status was a major factor that affected adherence among pregnant women [6–9, 13]. Other risk factors for non-adherence included a younger maternal age, lack of income-generating activity, early enrollment in the PMTCT services, experiences of discrimination, and lack of treatment support [6, 7, 9, 13].

ARV adherence is affected by the loss of patient follow-up [14, 15], and a loss to follow-up has been commonly observed in the PMTCT program in sub-Saharan Africa [16–18]. The effects of loss to follow-up on ARV adherence will become more serious in the longitudinal ARV regimen newly introduced in the WHO’s 2010 guidelines. However, few studies have examined longitudinal ARV adherence and the effects of the loss to follow-up.

Zambia is one of the countries seriously affected by HIV, with an estimated HIV prevalence of 12.6 % among the adult population in 2013 [19]. The national guidelines for PMTCT were revised based on option A of the WHO 2010 guidelines, which were implemented across the country at the time that the present study was conducted [20]. In accordance with the WHO 2013 guidelines, the Zambian government has been shifting to option B+, which is gradually being introduced at qualified heath facilities. In 2011, the majority of pregnant women (97 %) attended an antenatal clinic and underwent HIV testing, and 85 % of HIV-positive pregnant women received ARVs; however, only 36 % of infants born to HIV-positive women received ARVs [19]. The mother-to-child transmission rate was 12 % in 2012 [19]. However, the availability of HIV care/treatment services is limited. In 2010, there were 1784 health facilities in the country, and only 453 (25.4 %) offered HIV care/treatment services [21]. By 2012, about 110 facilities introduced HIV care/treatment services [19].

Before introducing the WHO 2010 guidelines, several studies have examined the uptake of ARVs among HIV-positive women and their infants in Zambia [22–28]. According to these studies, the maternal factors associated with a missed ARV dose were a longer interval between HIV testing and delivery [23], no high school-level education, lower newborn birth weight [24], maternal age between 26 and 30 years, multi-gravidity, fewer antenatal clinic visits, vaginal delivery, single-dose NVP regimen versus ART regimen [28], illiteracy, and no history of prior fetal or infant death [22]. In addition, the first diagnosis of HIV infection during pregnancy is a crucial factor that affects ARV adherence [29]. According to the new guidelines, ARV adherence and ART initiation can be affected by the availability of HIV care/treatment services, wherein PMTCT services are provided [30]. However, few studies have focused on these factors that affect ARV adherence in the PMTCT program in Zambia.

To date, no studies have examined the long-term ARV adherence under the 2010 national guidelines in Zambia. An evaluation of longitudinal ARV adherence would provide a platform to examine the feasibility of the WHO 2013 guidelines. This study aimed to assess ARV adherence from pregnancy to 24 weeks postpartum by considering the effect of loss to follow-up, and identify risk factors for non-adherence to ARVs.

## Methods

### Study setting

This prospective cohort study was conducted in Chongwe district, Zambia from June 2011 to January 2014. At the beginning of the study, there were 39 health facilities across the district. This study included the western part of the district as the study site where the Chongwe referral health center (HC) and 19 rural HCs are operated. All of the facilities have implemented the PMTCT program under the 2010 national guidelines, and only six provided HIV care/treatment. The Chongwe referral HC was equivalent to a district hospital, and it supervised the 19 rural HCs with regards to the implementation of the PMTCT and HIV care/treatment programs.

Of these health facilities, 11 were selected as the study sites. First, we selected all facilities that offered PMTCT services and HIV care/treatment, including one referral HC and five rural HCs. The HIV care/treatment was provided daily at the Chongwe referral HC and fortnightly at the five rural HCs. Then of 14 rural HCs, we selected five rural HCs that did not provide HIV care/treatment. At these facilities, women eligible for ART were referred to the Chongwe referral HC or one of the five rural HCs that provided HIV care/treatment. The five rural HCs with no HIV care/treatment were selected based on the following inclusion criteria: located within 20 km from a neighboring health facility offering HIV care/treatment, and delivery care was provided. Finally, 11 health facilities were included in the study.

### Data collection

HIV-positive women were recruited by nurses or trained volunteers when they attended PMTCT services from pregnancy (median 10 weeks before delivery) to the postpartum period (median six weeks after delivery). During the study period, we enrolled 360 women in the study, and analyzed 321 eligible women. Then, 39 women were excluded for the following reasons: 29 missed key data; eight had an abortion or stillbirth during pregnancy; and two were not breastfeeding at recruitment.

During the study period, participants had a maximum of four interviews at the following periods: during pregnancy (first observation), one week postpartum (second observation), six weeks postpartum (third observation), and 24 weeks postpartum (forth observation). The cutoff points for the follow-up observations were set at five weeks postpartum for the second observation, 23 weeks postpartum for the third observation, and 52 weeks postpartum for the fourth observation. All observations were completed either at the fourth observation, at the end of the interview survey (October 1, 2012), or at 52 weeks postpartum. The time points for the postpartum observations were decided by considering the timing of routine PMTCT visits [20].

Structured questionnaires were developed based on standardized questionnaires produced by the WHO [31], 2007 Zambia Demographic and Health Survey [32], and Adult AIDS Clinical Trials Group [33, 34]. Through face-to-face interviews, participants provided their age, educational level, marital status, type of health facility (referral HC, rural HCs with HIV care/treatment, or rural HCs without HIV care/treatment), travel time from home to the health facility, HIV status at enrollment in the PMTCT program, timing of study enrollment (pregnancy or postpartum), ARV regimen (ARV prophylaxis or ART), breastfeeding status, internalized stigma (i.e., feeling bad about being HIV-positive), and attitude about taking ARVs with three items (taking ARVs can prevent HIV transmission to the baby; taking ARVs makes me healthier; and if I do not take ARVs properly, the ARVs will not work). Agreement with the three questions was regarded as having a positive attitude towards ARVs.

As for the partners’ characteristics, participants provided their experience with couple HIV testing and counseling (CHTC), disclosure of their HIV status to their partner, HIV status of the partner, and experience with domestic violence (e.g., verbal abuse, physical abuse, and/or being forced to leave home) [35]. If a woman reported any of the three types of domestic violence, it was defined as having experienced domestic violence.

Using the PMTCT-related register, we assessed the HIV status and survival status of infants who were born to the study participants as of January 2014. During the study period, these infants were recommended to undergo HIV testing at six weeks, six months, 12 months, and 18 months of age at the study sites. We examined each date of HIV testing that the infants had ever undergone. If an infant had undergone HIV testing more than once, their latest HIV status was used in the analysis. However, we were not able to confirm the breastfeeding status at the time that the infants underwent HIV testing because a number of information was missing in the register.

### Definition of the study outcome

This study examined adherence to ARVs as the primary outcome. To measure adherence to ARVs, we used the self-report questionnaire developed by Adult AIDS Clinical Trials Group [36]. Despite the potential risk of overestimation, the self-report measurements have been validated [37], and they are widely used in resource-limited settings [5]. During the study period, we used the national PMTCT guidelines based on the option A of WHO 2010 guidelines [20]. Eleven HCs prescribed AZT for women not eligible for treatment during pregnancy and NVP syrup for HIV-exposed infants during the postpartum period under the PMTCT program, whereas triple ARV drugs for women on ART were prescribed from pregnancy to the postpartum period under the HIV care/treatment service program at the Chongwe referral HC or at the five rural HCs offering HIV care/treatment.

Corresponding to the national protocol, ARV adherence was measured differently between women on ARV prophylaxis and those on ART. For women on ARV prophylaxis, maternal adherence was assessed during pregnancy, and infant adherence was assessed during the postpartum period. For those on ART, maternal adherence was measured over the assessment period.

For both groups of women, non-adherence was defined as having missed any prescribed drug or having failed to follow the prescribing schedule at least once in the four days preceding the interview [7, 38]. Medication interruption was also defined as non-adherence if it was detected during the interview (primary definition).

For the women on ARV prophylaxis, the assessment of ARV adherence was completed on cessation of breastfeeding and confirmation of the HIV-negative status of their infants, as there was no longer a risk of HIV transmission to their infants. However, women on ART continued the assessment of ARV adherence regardless of the breastfeeding status, as they require lifelong ART.

Additionally, the authors expected that a number of participants would be lost to follow-up and would likely not adhere to ARVs during the period of loss to follow-up [15]. To include participants lost to follow-up in the analysis, we adopted a broader definition of non-adherence in which delayed or missed scheduled visits and reported infant deaths were treated as non-adherence (secondary definition).

### Statistical analysis

In the descriptive analysis, the chi-square test was performed to assess proportional differences in the categorical variables. The ARV adherence rate was calculated at four observed periods among the women who had attended the scheduled interviews, which did not include the women who had missed scheduled visits (primary definition).

The probability of remaining adherent to ARVs was estimated using the Kaplan-Meier method, which applies the secondary definition of ARV adherence using the time from the start of an observation to the detection of non-adherence to ARVs or interruption of ARV administration due to loss to follow-up (an event). However, study participants may repeat the events during the assessment period, and the observed record could contain interval-censored records. To analyze multiple events, the recurrent event model was used in the analysis [39]. In this model, the censorship point adopted the midpoint of the previous observation and the current non-adherence events (the main model), as a non-adherent event was likely to have occurred before it was detected [40]. Observations were also censored at the midpoint between the previous observation and the cutoff point of the present observation if a participant was lost to follow-up. Changes in the ARV regimen from ARV prophylaxis to ART were treated as a time-dependent variable.

The proportional hazards assumption was tested using a log-log survival plot and Schoenfeld residuals. The Cox proportional hazard regression model was subsequently tested to identify the risk factors for non-adherence to ARVs in a backward stepwise model-building procedure. The final model-building process assigned independent variables to two groups: those regarded as essential in the study (i.e., age, educational level, marital status, health facility type, and timing of HIV diagnosis) were retained regardless of their significance, and other potential confounders were retained if the *p*-value was <0.05. In the Cox proportional hazard models, the potential correlations within individuals were adjusted using robust variance estimates.

Sensitivity analyses were performed to test the effect of the time definitions and ARV regimen on the study outcome. Regarding the effect of the time definition, an alternative model was developed in which crude cutoff points were applied to censorship to estimate the time to detect a non-adherent event. Subsequently, we assessed any differences in the hazard ratios between censorship at the midpoint (the main model) and at the crude cutoff point (the alternative model). Similarly, adherence to ARVs among those on ARV prophylaxis and those on ART were analyzed separately to examine any differences in the hazard ratios between the two ARV regimens.

The statistical analysis was performed using Stata SE, version 12 (Stata Corp., College Station, TX).

### Ethical considerations

This study was approved by the Institutional Ethics Committee of the National Center for Global Health and Medicine, the Research Ethics Committee of the University of Tokyo, and Biomedical Research Ethics Committee of the University of Zambia. Written informed consent was obtained from all participants at recruitment. When a participant was found to have any physical or psychosocial problems (e.g., domestic violence) through the interview, she was referred to health workers for further follow-up.

## Results

### Basic characteristics of the study participants

Table 1 shows the basic characteristics of the 321 HIV-positive women. The referral HC enrolled 130 women (40.5 %); the five rural HCs with HIV care/treatment enrolled 106 (33.0 %) women; and the five rural HCs without HIV care/treatment enrolled 85 women (26.5 %). Half of the women (49.8 %) were recruited in the study during pregnancy. The median age was 29 years (interquartile range [IQR] 24–34), 105 (32.7 %) had achieved an educational level higher than primary school (eight years), and 272 (84.7 %) were married or living with a partner. The median time required to access the nearest health center was 60 min (IQR 30–90). About half of the women (49.5 %) were newly diagnosed as HIV-positive during the current pregnancy, and 154 women (48.0 %) were already receiving ART at enrollment to the study.

**Table 1:** Basic characteristics of study participants

Characteristics	Overall	Referral HC^a^	Rural HCs with HIV care/treatment	Rural HCs without HIV care/treatment		*p*-value^‡^
*N* (%)	321	130 (40.5)	106 (33.0)	85 (26.5)		
Age						
Median (IQR^b^)	29 (24–34)	30 (24–33)	30.5 (26–34)	28 (22–35)		
≤ 29	163 (50.8)	64 (49.2)	48 (45.3)	51 (60.0)		0.1
≥ 30	158 (49.2)	66 (50.8)	58 (54.7)	34 (40.0)		
Educational level						
≤ Primary	216 (67.3)	76 (58.5)	81 (76.4)	59 (69.4)		0.01
≥ Secondary	105 (32.7)	54 (41.5)	25 (23.6)	26 (30.6)		
Marital status						
Married/living with partner	272 (84.7)	112 (86.2)	95 (89.6)	65 (76.5)		0.04
Other	49 (15.3)	18 (13.9)	11 (10.4)	20 (23.5)		
Travel time to health facility						
Median (IQR^b^)	60 (30–90)	30 (20–60)	90 (60–120)	60 (35–120)		
≤ 59 min	134 (41.7)	84 (64.6)	21 (19.8)	29 (34.1)		<0.01
≥ 60 min	187 (58.3)	46 (35.4)	85 (80.2)	56 (65.9)		
HIV status at PMTCT enrolment						
Already known HIV status	162 (50.5)	68 (52.3)	68 (64.2)	26 (30.6)		<0.01
Newly diagnosed	159 (49.5)	62 (47.7)	38 (35.9)	59 (69.4)		
ARV regimen at study enrolment						
ARV prophylaxis	167 (52.0)	66 (50.8)	49 (46.2)	52 (61.2)		0.1
ART	154 (48.0)	64 (49.2)	57 (53.8)	33 (38.8)		
Timing of study enrolment						
During pregnancy	160 (49.8)	55 (42.3)	62 (58.5)	43 (50.6)		0.05
After delivery	161 (50.2)	75 (57.7)	44 (41.5)	42 (49.4)		
Baseline ARV adherence						
Adherent	254 (79.1)	102 (78.5)	89 (84.0)	63 (74.1)		0.2
Non-adherent	67 (20.9)	28 (21.5)	17 (16.0)	22 (25.9)		
Internalized stigma						
Yes	107 (33.3)	46 (35.4)	31 (29.3)	30 (35.3)		0.6
No	214 (66.7)	84 (64.6)	75 (70.8)	55 (64.7)		
Attitude on taking ARV						
Positive	278 (86.6)	114 (87.7)	90 (84.9)	74 (87.1)		0.8
Negative	43 (13.4)	16 (12.3)	16 (15.1)	11 (12.9)		
Couple HIV testing and counseling						
Received	129 (40.2)	33 (25.4)	60 (56.6)	36 (42.4)		<0.01
Never received	192 (59.8)	97 (74.6)	46 (43.4)	49 (57.7)		
Disclosed HIV status to partner						
Disclosed	258 (80.4)	109 (83.9)	88 (83.0)	61 (71.8)		0.07
Never disclosed	63 (19.6)	21 (16.2)	18 (17.0)	24 (28.2)		
HIV status of partner						
Positive	134 (41.7)	58 (44.6)	48 (45.3)	28 (32.9)		0.03
Negative	52 (16.2)	13 (10.0)	23 (21.7)	16 (18.8)		
Unknown	135 (42.1)	59 (45.4)	35 (33.0)	41 (48.2)		
Domestic violence						
Experienced	62 (19.3)	28 (21.5)	21 (19.8)	13 (15.3)		0.5
Not experienced	259 (80.7)	102 (78.5)	85 (80.2)	72 (84.7)		

^a^HC: health center

^b^IQR: interquartile range

^‡^ *p*-value for Chi-square test

During the baseline interview, 67 women (20.9 %) were non-adherent to ARVs. One-third (33.3 %) reported internalized stigma, and 278 (86.6 %) showed positive attitudes towards taking ARVs. Moreover, 129 women (40.2 %) had undergone CHTC, and 258 (80.4 %) had disclosed their HIV status to their partners. Of all the women, 134 (41.7 %) reported that their partners were HIV-positive, 52 (16.2 %) were HIV-negative, and 135 (42.1 %) had an unknown HIV status. Sixty-two women (19.3 %) reported that they had recently experienced domestic violence.

### Self-reported adherence to ARVs at four observation points

Table 2 shows the proportion of adherence to ARVs among the women who made the scheduled visits (primary definition). The ARV adherence rate was 82.5 % (95 % confidence interval [CI] 76.5–88.5 %) during pregnancy, 84.2 % (95 % CI 78.5–89.8 %) at one week postpartum, 81.5 % (95 % CI 76.1–86.9 %) at six weeks postpartum, and 70.5 % (95 % CI 62.3–78.7 %) at 24 weeks postpartum. At the 24 weeks postpartum follow-up, five women reported cessation of breastfeeding. However, they were included in the analysis as all of them were undergoing ART.

**Table 2:** Adherence to ARVs at four observation points^a^

Observation points	Overall	Adherent		
	*n*	*n*	%	95 % CI
Pregnancy	160	132	82.5	(76.5–88.5)
1 week postpartum	164	138	84.2	(78.5–89.8)
6 weeks postpartum	200	163	81.5	(76.1–86.9)
24 weeks postpartum	122	86	70.5	(62.3–78.7)

^a^Self-reported adherence to ARVs for the four days prior to the interview (primary definition)

### Probability of remaining adherent to ARVs

**Fig. 1:**
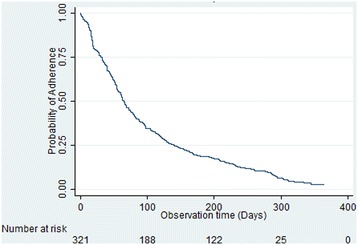
Probability of remaining adherent to ARVs. Definition of non-adherence to ARVs (secondary definition) includes: missed doses; not following prescribed schedule; missed visiting for scheduled interview; loss to follow up; and infant deaths

Figure 1 shows the probability of remaining adherent to ARVs in all participants, in which loss to follow-up and infant death were treated as non-adherence (secondary definition). The probability of remaining adherent to ARVs was 0.61 (95 % CI 0.56–0.66) at day 50, 0.35 (95 % CI 0.30–0.39) at day 100, 0.18 (95 % CI 0.14–0.21) at day 200, and 0.06 (95 % CI 0.04–0.09) at day 300.

### Risk factors for non-adherence to ARVs

Before running the Cox proportional hazard models, the proportional hazard assumption was evaluated using a log-log survival plot and Schoenfeld residuals, which showed no significance in the global test (*p* = 0.8). Overall, the model appeared to meet the proportional hazard assumption. Finally, the risk factors for non-ARV adherence using the secondary definition were estimated using the Cox proportional hazard models (Table 3).

**Table 3:** Risk factors for non-adherence to ARV

Variables	Main-final model	
	aHR^a^	95 % CI
Age		
≤ 29	1.00	
≥ 30	0.99	(0.82–1.19)
Educational level		
≤ Primary	1.00	
≥ Secondary	0.91	(0.75–1.11)
Marital status		
Married/living with partner	1.00	
Other	1.03	(0.80–1.34)
Facility type		
Referral HC	1.00	
Rural HCs with HIV care/treatment	0.71	(0.57–0.88)
Rural HCs without HIV care/treatment	0.58	(0.46–0.74)
HIV status at PMTCT enrolment		
Already known HIV status	1.00	
Newly diagnosed	1.24	(1.03–1.50)

Cox proportional hazard regressions

^a^aHR: adjusted hazard ratio

In the main final model, the women attending rural HCs providing HIV care/treatment (adjusted hazard ratio [aHR] 0.71, 95 % CI 0.57–0.88) and the women attending rural HCs not providing HIV care/treatment (aHR 0.58, 95 % CI 0.46–0.74) were more likely to be adherent than those attending the referral HC. Women newly diagnosed as HIV-positive during pregnancy (aHR 1.24, 95 % CI 1.03–1.50) were more likely to be non-adherent than those with a known HIV status before pregnancy.

### Sensitivity analysis

This study performed two sensitivity analyses to assess the following effects on the study outcome: 1) the time definition, and 2) ARV regimen. For the time definition, we utilized two models for the censorship definitions. The main model used a midpoint between the date of the previous observation and the cutoff points of the current observation, whereas the alternative model used a crude cutoff point. A comparison between the two models showed that the health facility type was a significant predictor in both models. Although the sensitivity analysis indicated that the definition of time affected the CIs of the HIV status at PMTCT enrollment, it did not show changes in the direction of the hazard ratios. Furthermore, a comparison between the two ARV regimens showed that the health facility type was a significant factor in both regimen groups, whereas the HIV status at PMTCT enrollment was a significant factor in the ART regimen group only.

### Mother-to-child transmission of HIV

Table 4 shows the preliminary results of the HIV testing among infants born to the study participants. During the study period, the participants had delivered 326 infants, including five sets of twins. Although the breastfeeding status could not be confirmed, 262 infants (80.4 %) underwent HIV testing at the median age of 10.4 weeks (IQR 6.5–28.6), which was the time that they had the latest HIV testing, and 11 (3.4 %) were diagnosed as HIV-positive by January 2014.

**Table 4:** Preliminary results of the HIV testing among HIV-exposed infants

HIV status	Number	Percent
HIV positive	11	(3.4)
HIV negative	251	(77.0)
Unknown	64	(19.6)

As of January 2014

## Discussion

In this study, the self-reported ARV adherence rate (primary definition) was over 80 % from pregnancy to six weeks postpartum, but it decreased to 70 % at 24 weeks postpartum. Using the broader definition, which treated the loss to follow-up as non-adherence (secondary definition), the probability of remaining adherent to ARVs decreased considerably over the assessment period. The risk factors for non-adherence to ARVs were identified using the secondary definition, which included attending the referral HC and having a new diagnosis of HIV infection during the current pregnancy. Potential confounders such as the timing of study enrollment and the ARV regimen (ARV prophylaxis/ART) did not affect the study outcome. The small sample size may be one of the reasons for this finding. Eighty percent of the infants had undergone HIV testing, and 3.4 % were HIV-positive, although their breastfeeding status was unknown.

This study observed poor longitudinal adherence to ARVs in the PMTCT program. Similar findings were also reported in studies conducted in other sub-Saharan African countries [5–9]. An extended ARV regimen was the major revision in the WHO 2010 guidelines [2]. The poor longitudinal ARV adherence highlights critical challenges to implementing the WHO 2010 guidelines. Another explanation for this finding would be that this study was conducted shortly after the introduction of the new guidelines; thus, the extended regimen may not have been familiar at the operational level.

In particular, women attending the referral HC showed poorer adherence to ARVs than those attending rural HCs. In previous studies, easier access to health facilities was found to facilitate better ARV adherence [10]. In this study, women attending the referral HC reported a shorter travel time to the health facility than those attending rural HCs, which did not seem to influence their adherence. Potential reasons could be a poor quality of PMTCT services provided at the referral HC, which would be attributed to a large number of PMTCT clients per health worker. For example, only a quarter of the participants attending referral HC reported that they attended CHTC, whereas >40 % attended CHTC at other rural HCs. The gap in CHTC attendance may affect their adherence to ARVs [41–43] and the loss to follow-up [26]. A higher number of HIV patients per health workers can also increase the risk of loss to follow-up at the facility [44].

Furthermore, in rural HCs, the limited availability of laboratory and clinical services did not affect women’s adherence to ARVs. This may be because the fewer number of PMTCT clients at rural HCs may allow health workers to provide effective adherence support. Similarly, a study conducted in Malawi reported that continuous follow-up by community health workers has contributed to utilization and retention in the PMTCT program [45].

Regarding the service provision system at the time of implementing this study, the women needed to be transferred to other health facilities with HIV care/treatment services. However, only 25 % of health facilities offered HIV care/treatment services in Zambia in 2010 [21]. Such limited availability of the HIV care/treatment could be an obstacle for the transfer of HIV-positive women to the HIV care/treatment, which would result in loss to follow-up and treatment interruption [46]. The Zambian government has been expanding option B+ across the country since 2013, and all target facilities of the study have introduced option B+ by August 2015. In option B+, women attending rural HCs without HIV care/treatment services will face the challenge of continuing ART even after completing the PMTCT program. To implement option B+ across the country, effective planning is required to expand the HIV care/treatment services to all rural HCs simultaneously.

In this study, the women newly diagnosed as HIV-positive were more likely to be non-adherent than those with a known HIV status before pregnancy. A similar finding was reported in a previous study [29]. This implies that women who are newly diagnosed as HIV-positive have specific characteristics. First, the newly diagnosed women have to handle the physical and psychosocial challenges such as giving birth to a child with a risk of HIV infection, disclosing their HIV status to family members, following the daily routine of medication, and experiencing the side effects of ARVs [10, 47]. Second, they may not completely understand the importance of ARV adherence because they are more likely to be diagnosed during the asymptomatic stage; thus, they are less likely to experience the efficacy of ARV drugs [29, 48]. Third, they may have some misconceptions or negative impressions of HIV treatment [47].

In newly diagnosed women, psychosocial support and health education would be essential interventions to overcome multiple challenges and establish good adherence to ARVs. Thus, it would be important to ensure referral of the newly diagnosed HIV-positive women to HIV care/treatment services where they are able to receive comprehensive care and support that may not be provided in the PMTCT program. However, only six facilities provided HIV care/treatment services at the study site, which is common in other parts of Zambia. This suggests that further effort is required to strengthen the linkage between the PMTCT and HIV care/treatment programs [46].

To mitigate psychosocial challenges following antenatal HIV diagnosis, women should be encouraged to undergo HIV testing before pregnancy. This will reduce the number of pregnant women who are not aware of their HIV status until the first antenatal visit. Furthermore, early awareness of their HIV status would encourage women to abstain from risky sexual behavior, which would reduce the risk of HIV transmission to their children and partners [49]. Moreover, this approach would provide the benefit of primary HIV prevention among the population of reproductive age. In this study, however, half of the participants did not know their HIV status, and 40 % did not know their partners’ HIV status. Similarly, nearly 70 % of HIV-positive men and 50 % of HIV-positive women in Zambia are reported to have never been tested before their first HIV-positive diagnosis [32]. Thus, the promotion of regular testing of all sexually active women of reproductive age would increase the benefits associated with PMTCT strategies.

This study has several limitations. First, the small sample size may limit the statistical power and affect the study findings, and it may affect the generalizability of our findings. Second, variation in the timing of study enrollment may cause selection bias even though we statistically adjusted and confirmed that the study outcome was not affected by the timing of study enrollment. Third, this study used a single measurement of self-reported ARV adherence, which was potentially affected by recall bias and social desirability bias. Forth, this study may have overestimated the incidence of non-adherence due to use of a broad definition of ARV adherence. However, this is an operational research using intention-to-treat analysis, which was designed to observe the standard practice of the PMTCT program. Finally, given the observational nature of this analysis, the results may be biased owing to the unmeasured confounders at the individual, facility, or community level.

## Conclusions

The PMTCT program in Zambia is facing a crucial operational challenge of poor adherence to ARVs over the high-risk period of mother-to-child transmission. All health facilities should strengthen the support for ARV adherence and retention in care from pregnancy to the cessation of breastfeeding.

## Abbreviations

HIV: Human immunodeficiency virus
WHO: World Health Organization
PMTCT: Prevention of mother-to-child transmission of HIV
ARV: Antiretroviral
AZT: Zidovudine
NVP: Nevirapine
ART: Antiretroviral therapy
HC: Health center
CHTC: Couple HIV testing and counseling
IQR: Interquartile range
aHR: Adjusted hazard ratio
